# Is Government Policy a Barrier or Facilitator to the Work of Place-Based Community-led Nonprofits?

**DOI:** 10.1515/npf-2024-0003

**Published:** 2024-12-05

**Authors:** Michael J. Roy, Jack Rendall, Emma Clewett, Julie McMullan, Rosie Read, Jennifer McLean, Liam O’Hare, Marcello Bertotti, Mike Kelly, Rachel Baker

**Affiliations:** 7622 Stirling Management School, University of Stirling, Stirling, Scotland; Yunus Centre for Social Business and Health, Glasgow Caledonian University, Glasgow, Scotland; Institute for Connected Communities, University of East London, London, UK; School of Social Sciences, Education and Social Work, Queen’s University Belfast, Belfast, UK; Bournemouth University, Poole, Dorset, UK; Glasgow Centre for Population Health, Glasgow, UK; University of Cambridge, Cambridge, UK

**Keywords:** nonprofits, social policy, government, abductive analysis, UK, public health

## Abstract

In this paper we explore whether government policy acts as a barrier or facilitator to the work of nonprofits, and in what ways (if at all) different policy contexts influence the work of place-based community-led nonprofit organizations working to improve the health and wellbeing of their communities. Employing ‘abductive’ analysis of qualitative data collected through in-depth semi-structured interviews and focus groups with 20 non-profit leaders located in different parts of the UK, we found that government policy can act both as barrier and facilitator at various times. There is often a disconnection between policy intent and the realities of local context, funding arrangements for nonprofits was often dependent on developing and maintaining successful relationships, and there were clear tensions between the pursuit of mission and alignment to government priorities.

## 1 Introduction

Over the last few decades, relationships between the non-profit sector and government policy have become increasingly understood (Bassi 2023). Many of the actions of nonprofits, of their management and trustees, are enabled or constrained by legislative requirements specific to where they operate, and nonprofits regularly advocate for government support, often in the form of funding, to further their social mission and local impact (Fyall 2016). It has therefore become increasingly axiomatic to state that it is “impossible to understand the formation, operation, and management of nonprofits without a commensurate understanding of the public policy context they inhabit” (Vaughan and Arsneault 2013: 2). While international cross-comparative studies of nonprofit organizations exist – indeed was the focus of the seminal Johns Hopkins Comparative Nonprofit Sector Project1https://ccss.jhu.edu/research-projects/comparative-nonprofit-sector-project/. – studies that adopt a granular “street level” perspective (viz. Lipsky 1980) of the direct interactions between nonprofits and their ‘clients’ – on whether (and how) the work of government supports or inhibits such work – are still relatively scarce, particularly outside of the United States (de Morais Holanda, Kim, and Appe 2023). Empirical studies which draw upon data from nonprofits across different policy jurisdictions from such a granular perspective, especially in relation to the post-COVID environment, are rarer still (although see Rees et al. 2023).

This paper focuses on the notion of policy context, extending knowledge of how and why it matters, and how policy inhibits or supports the work of nonprofits engaging in place-based activities focused on improving the health and wellbeing of their immediate communities. For the purposes of this paper, we define policy broadly as government action, including what policymakers *say* they are going to do, as well as what they *actually* do. We are concerned with both elected and unelected policymakers, as well as the individuals, groups and organizations that help influence and deliver policy outcomes, including at the ‘street level’ (Cairney 2019). Thus ‘policy context’ is understood as the ‘complex environment’ in which such government action plays out, particularly as a result of interactions between stakeholders “where decisions about policies are discussed and, most importantly, where they are implemented” (Echt 2017). Our study is part of a larger project called Common Health Assets,2https://www.commonhealthassets.uk. focusing on the role of local ‘community-led organizations’3All the ‘Community-led Organisations’ we worked with within this project are companies limited by guarantee with charitable status under UK law, analogous to US nonprofit organizations governed by the 501(c)3 exemption. We are therefore confident about using the term ‘nonprofits’ throughout this paper despite this not being the dominant terminology in use in the UK. and their health and wellbeing impacts. In this project (see Baker et al. 2023) realist evaluation (Pawson and Tilley 1997) has been adopted. Over the last decade or so realist evaluation method has become increasingly common, especially in public health research, to assess particularly complex forms of ‘intervention’ (Craig et al. 2019; Skivington et al. 2021). However, it is still far from commonplace in policy research. We can conceptualize nonprofits, and the activities they deliver, as highly complex forms of ‘intervention’ since they are dependent on interactions between many component parts, both internal and external to the organization (Roy et al. 2014), operate in open, rather than closed, systems, and regularly involve a variety of feedback loops. Moreover, complexity is also a key property of the community context (or system) in which the nonprofit is embedded (viz Hawe 2015).

This study constitutes an analysis of the issues viewed from the ‘ground up’ in organisations in various parts of the UK to feed into a realist understanding of context and the ‘contextual mechanisms’ at work (see Porter 2015). We did not undertake a comparison of the non-profit experience in three of the devolved nations of the UK; it was not a systematic comparative investigation. We focus attention on nonprofits working in so-called ‘deprived’ communities (according to indices of multiple deprivation) with a particular focus on place-based community-led nonprofits working on improving community health and wellbeing, based in England, Scotland, and Northern Ireland. Many aspects of policy that impinge upon nonprofits are devolved from Westminster to the parliaments in Edinburgh and Belfast respectively, and there is evidence of policy divergence in a significant number of areas (Keating et al. 2003; MacKinnon 2015), including in relation to the COVID response (Acheson et al. 2022).

We were guided by the following research questions: (1) *How does policy act as a barrier or facilitator to the work of nonprofits?* and (2) *In what ways do different policy contexts influence the work of nonprofit organizations, if at all?* Our paper is organized as follows: after a discussion of the relevant background literature, we turn attention to the methods that guided the study, including our analytical processes. We then discuss the findings in the context of extant literature, before reflecting on our contributions, both from the perspective of scholarship, and for policymakers. First of all, however, we consider: why does government policy matter to the work of nonprofits? And what do we know about how policy context can positively or negatively influence their work?

## 2 Background: Why Does Policy Matter to the Work of Nonprofits?

The groundbreaking work of Lester Salamon and many others since has shown that there is an ‘interdependence’ between nonprofits and government (Salamon 1995). Contrary to a widespread belief that with the development and scaling up of modern welfare states the voluntary and community sectors would be ‘crowded out’ and shrink in size to accommodate, in fact the sector has grown in size and importance commensurately (Salamon and Anheier 1996; Salamon and Toepler 2015). The state not only makes extensive use of the nonprofit sector to deliver services, but also finances a great deal of nonprofit activities (Salamon 1995). However, as Shafiq, Albrecht, and LeRoux (2023) has recently pointed out, the vast majority of work that has utilized Salamon’s theory of interdependence has depended upon government data on partnerships between nonprofits and the state, rather than data from the nonprofits themselves. These data also tend to be at a significantly higher level of abstraction than the ‘street level’ perspective of direct nonprofit and client interactions we adopt in this study.

We acknowledge that there is a voluminous literature on various forms of partnership working between nonprofits and the state (Bode and Brandsen 2014; Zimmer 2010) including a recent focus on collaborative forms of governance (Ansell and Gash 2008) and – most relevant to this study – the role of nonprofits within those (Brandsen and Johnston 2018; Calò et al. 2024). The literature on ‘co-production’ processes that involve both users and public sector professionals in the design and delivery of public services (Nabatchi, Sancino, and Sicilia 2017) tells us that close and effective working relationships between nonprofits and policymakers can result in positive outcomes for communities (Bovaird 2007; Brandsen and Pestoff 2006). However, there is not only considerable ambiguity and contestation on what constitutes ‘co-production’ (Nabatchi, Sancino, and Sicilia 2017) but *too* close a relationship between government and nonprofits can potentially lead to isomorphic tendencies (viz. DiMaggio and Powell 1983) where nonprofits can come to resemble – for better or for worse – government bodies (Leiter 2008; Rees, Taylor, and Damm 2024).

There is also an emerging literature on how the nonprofit sector worked to support communities during the COVID crisis and the nature of their relationships with government during that time. Rendall et al. (2022), for instance, drew upon Young’s (2006) important work which noted that relationships between the sector and the state were complementary, supplementary, and/or adversarial at various times throughout the crisis. Recently acknowledging the ‘adaptive entrepreneurial culture’ of many nonprofits during this time, Young (2023: 237) reminds us that:

The view of nonprofits as resilient institutions has been reinforced since by their performance in recent crises including the COVID pandemic beginning in 2020 and the financial crisis of 2008–2009, though not without exception or assurance that nonprofits would necessarily be resilient in future crises.

Cullingworth et al. (2024) expressly go further than Young’s schema, emphasizing a potential ‘co-pilot’ role of the nonprofit sector. In fact, during the COVID pandemic the state was almost completely dependent upon the nonprofit sector for a time, even for the provision of statutory services. We know that during crisis situations – during wars, as well as pandemics – (see Kabalo and Almog-Bar 2023) close working between the state and nonprofits is seen as vital, but without clear appreciation of the local contextual factors that facilitate or inhibit close working, it is difficult to understand what needs to be in place to allow this to happen. We recognize too that traditional theories of state-nonprofit relations are generally presented as relatively stable, or static. The COVID-19 pandemic upended this idea: Bennett et al. (2024) have argued for a more dynamic and nuanced understanding of state-nonprofit relations in their examination of smaller nonprofit human service organizations operating in England and Wales during the pandemic.

One important element of policy context relates to funding. Since at least the New Public Management reforms of the 1980s (Dunleavy and Hood 1994), the nonprofit sector has become increasingly central to the delivery of government programs, with a noticeable shift in recent times from grant funding to the state procuring goods and services from the nonprofit sector under contract. Nonprofits are increasingly expected to submit competitive tenders and, in many cases, vigorously compete with the private (for profit) sector for such work (Barraket, Keast, and Furneaux 2015; Roy et al. 2024). The implications, including unintended consequences, of introducing market behaviors into the nonprofit sector has concerned academics for some time. While evidence on whether government funding suppresses the important political advocacy role of nonprofits (for instance) is mixed at best (Chavesc, Stephens, and Galaskiewicz 2004), it is clear that the marketisation of the nonprofit sector (Alcock and Kendall 2011; Carmel and Harlock 2008; Dayson and Wells 2013) has had a transformational role on the sector as a whole (Evans, Richmond, and Shields 2005; McKay et al. 2015; Roy, Eikenberry, and Teasdale 2022). The closeness of the nonprofit sector to the state – its relative inability to ‘bite the hand that feeds it’ (Cullingworth et al. 2024) – has raised questions over the independence of the sector, potentially placing the important role of civil society to place checks on both government, and the market, at risk (Dart 2004; Egdell and Dutton 2017; Eikenberry and Kluver 2004).

Another contextual factor, and a key consideration for this study, is the policy contexts in which nonprofits operate. These landscapes can differ significantly across countries, regions, municipalities, and communities. As this study is focused on the UK and compares England, Northern Ireland and Scotland, several factors influence the political/policy landscape that are worthy of note. Despite the UK ostensibly being a ‘union’ of four different countries, the devolution arrangements are highly uneven and ‘asymmetrical’ (MacKinnon 2015) with different countries having different powers to varying degrees. While power is still highly concentrated in Westminster (Curtice 2006; Keating, Cairney, and Hepburn 2009); the constituent countries of the UK have less autonomy than most states in the US. Nevertheless, the devolution of powers that began in the late 1990s has created different policy landscapes for England, Wales, Northern Ireland and Scotland (Acheson et al. 2022; Alcock 2012; Chaney and Wincott 2014) and policy divergences occur quite regularly. In the devolved administrations, nonprofit leaders regularly talk of feeling comparatively close to government decision-makers, and thus better placed to influence policy. The shared borders between Northern Ireland/Republic of Ireland and England/Scotland complicate the policy landscapes further. These policy landscapes are complex and continually evolving. One other complex political aspect that emerged during our study and thus deserves mention, was the outcome of the Northern Ireland Assembly elections of 2022. This was the first occasion that a nationalist party (Sinn Féin) became the largest in Stormont, but during the period of our fieldwork there was no power-sharing arrangement and thus no sitting government in Northern Ireland. This was ostensibly due to a disagreement between parties over a post-Brexit trading issue. Northern Ireland was essentially run by civil servants until February 2024, when the impasse was broken, and the Northern Ireland Assembly was able to reconvene.

For additional insight into the contemporary policy landscapes across England, Northern Ireland, and Scotland (our study did not include Wales, which has its own Assembly), we constructed a framework of nonprofit-relevant policies in each jurisdiction (which is not exhaustive), which can be found online.4https://www.commonhealthassets.uk/_files/ugd/f25c8a_c1affc22bdaa443099c65a193e9c19b0.pdf. We next turn attention to the methods employed during our study.

## 3 Methods

We had previously identified nonprofit organizations located in each of our study locations, namely Northern Ireland, Scotland, and England, who were willing to work with us for the three-year duration of the wider Common Health Assets project. For this phase of the research, we approached managers within each organization and conducted in-depth semi structured interviews (*n* = 12) and two mini focus groups involving four people each at their place of operations, thus we were able to draw upon comments from 20 individuals in total. Interviews varied in length from 34 min to 1 h 20 min in length, and our sample breakdown is shown at Table 1.

**Table 1:** Interview participants.

Participant code	Area	Date of interview	Position
L1	London	19/10/2022	Director of Knowledge and Innovation
L2	London	19/10/2022	Delivery Manager
L3	London	19/10/2022	Project Manager
L4	London	19/10/2022	Director
B1	Bournemouth	04/11/2022	Chief Executive Officer
B2	Bournemouth	18/07/2022	Marketing Coordinator
B3	Bournemouth	09/06/2022	Director of Services
B4	Bournemouth	04/05/2022	Artistic Director
B5	Bournemouth	04/05/2022	Chief Executive
B6	Bournemouth	12/07/2022	Operations Manager
B7	Bournemouth	12/07/2022	Director of Partnerships
B8	Bournemouth	17/05/2022	Project Coordinator
B9	Bournemouth	19/05/2022	Project Coordinator
S1	Scotland	20/01/2022	Manager
S2	Scotland	20/01/2022	Manager
S3	Scotland	20/01/2022	Manager
S4	Scotland	20/01/2022	Manager
NI1	Northern Ireland	25/08/2022	Project Manager
NI2	Northern Ireland	23/08/2022	Manager
NI3	Northern Ireland	23/08/2022	Manager

As such, this represents a form of ‘purposive’ sampling (Mason 1996) given that they were both willing to work with us, and that we were confident of rich, relevant data from all. As this was a multi-site project involving a range of different research partners, ethical approval was sought from the relevant ethics committee at each of the four universities involved at the start of the project. To expand our understanding of policies as they impact at ‘street-level’, the interview questions framed ‘policy’ in an open-ended manner in line with our broad definition set out above, allowing participants scope to reflect on the impact of policies at both a general and more specific level, as they apply to and are experienced within their area of practice. We focused on the experiences of relationships with policymakers, whether they had to adapt their delivery based upon changes in local or national policy, and the relationship – if any – between policy and funding. We recorded each interview ‘intelligent verbatim’ (see McMullin 2023) and imported the raw data into the computer-assisted qualitative data analysis program NVivo 12 (Bazeley and Jackson 2013) to help structure our coding and analysis.

After an initial round of open coding (viz. Saldaña 2013), we coded and organized our data thematically (Braun and Clarke 2006). We did not construct our themes *a priori*, nor purely inductively. Instead we undertook a form of abductive (Peirce 1932; Timmermans and Tavory 2012) inference, where explanations for findings are generated “by moving backward and forward among empirical data, research literature, and emergent theory” (Dey and Teasdale 2013: 255) thus allowing any unexpected or divergent results to emerge. This ‘emergent’ form of analysis has been presented according to the various themes we identified and structured around our research questions; our researchers generated an initial list of first-order codes from the dataset that had a reoccurring pattern, then searched for recurring patterns, linkages, categories, and subcategories within the first-order codes relating to each research question. The whole process continued in parallel with data collection until theoretical saturation occurred and no further themes emerged. Instances of disagreement were resolved through discussion, a process that continued until we had agreement and confidence in the theoretical validity of the emergent themes.

We next turn attention to our findings, organised into five main themes, namely: (i) Disconnect between policy and the realities of context; (ii) Sustainability and funding; (iii) Tensions between the pursuit of mission and alignment to government policies; (iv) Implications for policy innovation; and (v) Relationships between nonprofits and policymakers/commissioners.

## 4 Findings

### 4.1 Disconnect Between Policy and the Realities of Context

Some participants expressed the view that there is a significant disconnect between policy and the reality of service delivery. Although there may be good intent, there was a feeling within nonprofit organizations of a lack of trust in communities by those developing such policies:

Government policy blows… it’s like a pendulum… community will be there forever and so you’re constantly having to interpret government policy in a way that makes sense to communities, and hopefully in the other direction, trying to help governments understand communities… lots of mismatch between policy and what happens on the ground… policy needs to understand what issues are driving the needs of the community. (L1, Director of Knowledge and Innovation)

That said, there was also a general perception that the engagement of community organizations in policy development was highly significant to the public sector. The nonprofits that we interviewed all explained that they were continually engaged in policy processes to varying degrees. The ‘lived experience’ they could impart was felt to be encouraged and valued by policymakers. However, the process of engagement is regularly laced with complexity in terms of gathering and translating experiences:

We [community organizations] are connectors that make policy real … Little did I know I was going to become a professional jigsaw player, because you have to get all the pieces out there, what communities are telling us, what do we know ourselves, what are other colleagues… and community organizations are telling us… what politicians are saying… and you put it on the table and you make some sense of it. (S2, Manager)

It can be difficult for policies to reflect local needs without hearing local voices, which many participants state is a regular challenge:

There would definitely be advantages of engaging with people on the ground, but at the same time I don’t think there is a solution for that because how do you do that in a meaningful way that isn’t tokenistic, and fits everyone?

I don’t know what the answer is… because the other way to do that is to just have an open consultation and everyone comes… then you have to decipher information from that. (L3, Project Manager)

Interpreting ‘policy language’ can be difficult for nonprofits and the communities they involve in their work. Many participants stated that policy is not written in lay language; if policy makers are encouraging wider community engagement, language should be more accessible at and relatable at a community level:

The language used in policy can be confusing, better communication is needed… it is not easy to digest the language. (L2, Delivery Manager)

Several respondents also stated that interpretation can be a challenge due to jargon and lack of resources to support engagement:

The policy is already written possibly, but then it is taken out to the community and asked for an opinion… by that time it may be difficult for someone to understand. For example, someone who has worked, say 20 years at community level at a local foodbank engaging with people in a client facing role… many don’t have experience working with policy so it can be difficult to interpret. (L2, Partnerships Manager)

Therefore, those in client-facing roles and individuals within communities can feel alienated by some of the terminology used within policy processes. One participant stressed the importance of two-way communication to ensure policies are responsive to local needs. Service delivery can inform the policy context which in turn, can influence the strategy of an organization:

What you’re delivering on the ground has to be relevant to government policy, it has to be seen to be answering an issue. So, those polices drive your organization’s strategic direction twofold, it comes back to that model of communication where it’s from top down and bottom up. In order for the policy to be responsive to the community, the sector has to be at the table to tell you what’s happening… so that your policy is representative of what’s needed. (NI1, Project Manager)

### 4.2 Sustainability and Funding

Two respondents from different cities mentioned that the impact of funding changes at national, regional, and local level can significantly affect nonprofits working locally. For example, nationally imposed cuts in mental health treatment centers had meant that more people were attending community organizations, thus significantly increasing demand for support, both in number of people, and in terms of the severity of mental health issues faced in communities. Another respondent reflected on the impact of national welfare policies on communities which can multiply demand for support almost overnight:

We will do a piece of work with two or three organizations locally that may be very successful, and then the next thing… that organization is bidding against us for our contract. That’s gone horribly … sometimes. (B3, Director of Services)

Money can be removed at any time. Our communities are absolutely at the mercy of government policy. I mean, [Professor Sir] Michael Marmot gave a fantastic example a couple of months ago of the government’s decision to withdraw the £20 a week uplift to Universal Credit. (L1, Director of Knowledge and Innovation)

As a result of the decision to withdraw the £20 uplift to Universal Credit (introduced during the pandemic), 100,000 more people in London alone found themselves in poverty, including more than 30,000 children (London Assembly 2021). This had immediate and long-term impacts on the operations of nonprofits dealing with the local community effects of this national policy change. Moreover, ‘machinery of government’ changes can disrupt relationships between nonprofits and policymakers due to structural and staffing changes. For instance, the merger of three local authority functions into one led to one of the nonprofits to report difficulties in maintaining relationships and associated access to funding and knowledge about emerging policy priorities.

Additionally, a policy commitment to addressing the factors in the social environment that can favor or harm health can be undermined by other government policy agendas, particularly those introduced under the ‘austerity’ regime in place in the UK from 2010 (Walsh, Wyper, and McCartney 2022). This includes the highly controversial ‘bedroom tax’ which aimed to reduce public spending on social housing by penalizing tenants living in housing deemed too large for their needs. This policy has had detrimental impacts on health and wellbeing and social relationships within homes and communities (Moffatt et al. 2016). This also includes the aforementioned cuts to benefits such as Universal Credit after the pandemic, and a wider environmental culture of ‘permacrisis’ in public spending for local government, and social care within that context. Such factors reduce the ability of local authorities to engage meaningfully and sustainably with potential innovations in non-clinical health service developments such as ‘social prescribing’ (Bertotti et al. 2018) which have direct relevance to many community-led and -based nonprofits.

Changes from direct award of funding to nonprofits to competitive tendering as well as the declining levels and short-termism of funding were significant issues for non-profit respondents who emphasized that the funding policy context has changed over time. These tend to be short-term, and can clearly affect the nonprofit in many ways (albeit this does not mean that those working in nonprofits do not have agency):

I think organizations, and to some extent communities, have much greater liberty to operate almost irrespective of government policy… they have much more ability to do that than they acknowledge for themselves. (L1, Director of Knowledge and Innovation)

One of the respondents mentioned that a ‘lot of funding’ was available in the late 1990s alongside a building where people could go to undertake theatrical acting. However, the situation gradually changed from 1999 to 2018 with a marked decline in funding available to deliver [community art] activities, creating a long-term problem:

Not just short but long-term problems as a result of cuts to the [local] Centre for Community Arts. But also lack of safe space to go to. 60 years of drama practice and arts practice cut. Then the advisory teachers cut, the Theatre in Education team cut, … [all] had a massive impact. (B4, Artistic Director)

Changes in funding regimes and funding levels have meant that nonprofits often need to spend substantial time, capacity, and resources in applying for funding, even for small grants from the local authority, sometimes to the detriment of delivering services:

So, there are small, local council pots, but it’s all little bit here, a little bit there. But we have to pay £100K in rent and all costs are going up. (B6, Operations Manager)

In addition to this, the short-term nature of funding was a critically important issue:

In most cases, it is three years funding. If lucky, five years. Say you get £1m. It takes you 12 months to set things up, one year to deliver, and then you need to start preparing yourself for the next tender over the last 12 months of three years funding. (B6, Operations Manager)

Importantly, the short-term nature of funding also led the respondent to conclude that there is not enough time to assess whether the funded intervention is actually working and learning to improve. Tendering arrangements were also described as a barrier, often leading to negative outcomes, such as local nonprofits going ‘out of existence’. In addition to this decline in funding (or perhaps because of it) competition between nonprofits has increased. Respondents highlighted that while they competed at the national and local levels for funding, competition for funding with other nonprofits can prevent cooperation and collaboration between them:

We want to collaborate, but we are all, even though we’re voluntary sector and we’re charities, these days it’s all about funding, you know. You gotta get your funding from somewhere. You don’t fundraise by rattling a tin in the supermarket anymore. (B6, Operations Manager)

### 4.3 Tensions Between the Pursuit of Mission and Alignment to Government Policies

When respondents were asked what facilitated their access to funding, an important theme emerging from respondents across the different jurisdictions was the strong alignment between meeting government policy and securing funding for their activities:

If you can show how you are meeting local or national policy guidance then that puts the organization in a stronger position for a successful bid. What follows policy is money. (S1, Manager)

[Our programs] must be aligned with policy in order to receive funding … from a funder/policy relationship it’s [about] trust: that we’re aligned to the same sort of values and that we deliver what we say we’re going to deliver. (L1, Director of Knowledge and Innovation)

Alignment is facilitated by ongoing trusting relationships between the nonprofit, community members and leaders, commissioners, and policymakers. Some respondents recognized that policies can also help to shape programs within the nonprofit by providing a focus on local needs, which can feed into the overall strategy of the organization, while others highlighted a clear tension between alignment to government priorities and the pursuit of the mission:

Neighbourhood Fund and other large grants are very useful. It’s fantastic that we got the money, but it also sends us in different directions as well and takes us away from our core work. Grant funding sets you off on a tangent because you have to fulfil the criteria if you want to get the money. (NI1, Project Manager)

The same point was reinforced by a respondent from Bournemouth in England who admitted that the priority of their nonprofit has to be on delivering projects they have had funded. One of the implications of this is that priority is almost always given to deliver funders’ (often government) aims and objectives, rather than what may be most needed by the communities they were set up to serve. Other respondents were more pragmatic about the tension between funders’ priorities and their mission, pointing out that the work of their nonprofit continues to be primarily driven locally and informed by their community. Their work may be positioned within a specific policy context, but is not dictated by this:

If we identified a particular need, we’ll look towards a policy that would shape or influence that and then seek the funding for the project to address it… so we always aim to start at the point of need and then look for a policy that fits that and a funding stream that fits it. (S2, Manager)

### 4.4 Implications for Policy Innovation

Despite the issues faced by nonprofits to adapt, mitigate and balance tensions with policy makers and commissioners, respondents also focused on a range of positive experiences and opportunities arising from their work with policy makers and commissioners. Where there was a good match between policy priorities and nonprofits’ expertise and strong links with communities, opportunities tend to emerge for nonprofits to work innovatively. Specific examples of this include recent health policy developments, notably ‘social prescribing’ arrangements where, if appropriate, people are empowered to find non-medical arrangements that will improve their health and wellbeing, often using services provided by the voluntary and community sector (Bertotti et al. 2018); health and social care integration (Strokosch and Roy 2024); and greater personalization of health and social care provision. Two nonprofits based at the London and Bournemouth sites respectively were strongly involved in these areas of practice. For example, the London-based respondent saw social prescribing as a policy area that can connect the health service to communities in new ways. Similarly, a Bournemouth nonprofit interviewee explained that:

A positive policy from our perspective might mean that there is investment in the local community through the local authority and the NHS [National Health Service] and we get to be part of delivering that or we get to be the provider of that service. (B3, Director of Service)

The social prescribing agenda was “about diverting people who did not need primary care services to more community-oriented services” (B3, Director of Service), thus alleviating pressure on more-acute parts of the system.

One respondent stated that in recent years a wider range of organizations had been invited to influence the policy around health and social care integration, supporting evidence that co-production of policy design between the nonprofit and public sectors is increasingly being encouraged. Reports developed by nonprofit organizations are often cited within government policy, particularly relating to healthcare.

Policy development and service delivery appear to have the opportunity to work positively in tandem, which can be advantageous if such communication is encouraged and resourced. Community-based nonprofit organizations have detailed grassroots knowledge of their communities and their needs, but barriers can be erected due to government designing policies without consulting, involving or engaging positively with nonprofit organizations:

We can provide on the ground day to day information and up to date knowledge that’s needed to guide and transform polices that affect the people we work with. (NI2, Project Manager)

One respondent suggested that the role of nonprofits was thus one of balancing policy priorities of commissioners with the needs of the community:

Government policy kept going backwards and forwards and our role was to cut a line down the middle… pick off points where it intersected with what was important in our community… and take advantage of government policy. (L1, Director of Knowledge and Innovation)

The government response to the pandemic was seen by most respondents as an important opportunity to highlight what is possible with close partnership working between the nonprofit sector and government. One respondent reinforced this point by describing the partnership working between their nonprofit and the local authority during the pandemic: “It had to be a partnership. It had to be really collaborative” (S2, Manager)*.* They described how they had to work as a team, and how moving towards a more collaborative relationship had helped them to better understand the needs and priorities of both. One respondent extended this point about partnership beyond the pandemic. In recent years, they felt the relationship between the local authority and the local community had improved due to better relationships with the nonprofit; there was now a more “co-produced and relationship-oriented conversation with people” (B3, Director of Services).

Further, the pandemic provided an opportunity for nonprofits to prove that they can quickly pivot and adapt; they had capacity and assets that could swiftly be employed for community benefit:

For us, COVID brought us more to the table because it allowed people to see how quickly we could adapt… the councils came to the community and voluntary sector to do it. (NI1, Project Manager)

They also experienced a marked improvement in responsiveness during the pandemic when they were granted permission by funders and policymakers to respond at pace to events and needs:

During COVID everything became so fast, everything was expedited, which was great… imagine how responsive we can be doing what we know is coming down the road… the mental health epidemic. (NI1, Project Manager)

### 4.5 Relationships Between Nonprofits and Policymakers/Commissioners

Professional relationships need to be considered carefully in relation to the work of nonprofits and policymakers and commissioners of services. Many of the those interviewed mentioned the importance of effective ongoing relationships and acknowledged the difficulties that can often arise when trying to communicate and work within the parameters set by policy. Furthermore, nonprofit managers pointed out that being timeously aware of policy changes is also linked to the quality of relationships, and recognized the importance of being viewed as trusted partners and the benefits this can bring to the day-to-day operation of the nonprofit.

The importance of intermediary organizations, such as the ‘third sector interfaces’ as seen in Scotland – a Scottish Government initiative designed to provide a single point of advice and contact in each local authority area – was highlighted as a vital conduit. Intermediaries can play a vital role in ensuring that nonprofits are able to keep up to date with policy changes and developments and formulate policy advocacy approaches. The effectiveness of advocacy is generally down to maintaining strong working connections between key individuals. Respondents highlighted a range of issues with establishing and maintaining such relationships, and the impact of perceived power imbalances: meeting with policymakers is usually on their terms, often with little notice, which can place huge demands on the nonprofit. Policymakers are viewed as wanting the expertise of the nonprofit and ‘access to communities’, but will rarely resource access to that adequately, if at all:

They want to access the resources and the knowledge and the learning from it. So yeah, that’s a big dichotomy for us. (S2, Manager)

These power imbalances not only manifest when nonprofit staff are expected to make themselves available but can even impact on what they are allocated in terms of funding. This led one of the respondents to believe that the distribution of resources is more to do with personal relationships than with the policy in question:

depending on who’s in power, there’s a bit of tension. Sometimes we get more and sometimes we get less, depending on the personality rather than the policy, even though the policy is there. (S2, Manager)

Finally, high turnover of staff within government and government restructuring, especially at a local level, can make it difficult to develop and maintain the relationships that are necessary to ensure successful partnership working.

## 5 Discussion

Our findings extend existing scholarly understanding of the engagement of nonprofits with policy in several ways. First, nonprofit leaders reflected upon policies from the perspective of what they saw as their organization’s core mission to serve the needs of a given community (however defined and located) and reflect its interests. The common attribute of policies that were seen by our research participants as facilitating their work were those perceived to be furthering their connection to, and work for, their communities. Nonprofit leaders spoke of the vital role of nonprofit organizations within communities and the need for policies and policymakers to understand community needs and highlighted the role of the nonprofit in facilitating this dialogue to take place. Similarly, those nonprofit managers who felt that they were trusted to influence policy development also tended to view it more positively facilitating their work; a good example being the trust and greater degrees of freedom that nonprofits seem to have been granted by policymakers and funders to respond quickly to local need and develop support for communities during the COVID-19 pandemic. Where nonprofits could readily align their work with policy objectives, they were better positioned to benefit from financial support. In terms of specific policy areas - nonprofit managers saw specific policy areas such as ‘social prescribing’ and ‘health and social care integration’ as positive policy developments they could influence directly, since their success critically relies upon the expertise and connections that nonprofits have with communities. That said, there is an argument that many nonprofits feel the need to align with government policy initiatives to survive, potentially at the risk of stifling local innovation, responsiveness, and effectiveness.

By the same token, nonprofit managers tended to define policies as barriers in instances where those policies failed to take account of the interests or needs of communities, at least as understood by them. Policies designed without consulting the nonprofit or the wider community they represent, or which take no account of local circumstances or the resources available to nonprofits to deliver outcomes required, were identified as barriers. Equally, the short-term nature of funding, as well as the non-availability of funding for the core work of some nonprofits, were seen as important obstacles to their work.

Second, our study shows that policies not directly related to nonprofits nonetheless can have a profound impact on their work and can be experienced as a facilitator or a barrier. Cuts to mental health budgets or social security benefits can result in rapid increases in need within communities and demands on nonprofits for social, emotional, and financial support. Public sector austerity has had a profound impact on the nonprofit sector in the UK. Although austerity bleeds through everything in terms of social inequalities, devolved governments and local authorities could, arguably, work more flexibly across public health and the nonprofit sector than they have been to date. They could work to deliver more stable, multi-year funding arrangements, for instance.

We saw evidence of the ability of government to be more flexible during the COVID crisis; indeed, another important finding was the substantively different relationship between government and nonprofits during the COVID-19 pandemic. Nonprofit organizations were recognized as partners who could deliver efficiently due to their agility and embeddedness in a given community (Bynner, McBride, and Weakley 2022). The level of cooperation and coordination between government and nonprofits increased substantially alongside the ability of the nonprofit sector to influence the way services were delivered (see also Kabalo and Almog-Bar 2023) and the bureaucracy involved in accessing funding was significantly relaxed for a time. Interestingly, respondents from across the three nations in question had similar experiences with government departments during this period, suggesting that during a period of crisis, the importance of working together across sectors to support the needs of communities should take precedence. This different relationship between government and nonprofit organizations was not just confined to the experience of respondents to this research, but was echoed in an evidence review (see Scottish Government 2022) which highlighted how lower bureaucratic burden and increased collaboration during the pandemic increased the flexibility and responsiveness of nonprofit organizations. This has also been recognized in the academic literature. Cullingworth et al. (2024: 104) explain that:

The sector’s ability to respond was, in part, enabled by the state, which made resources available, allowed organisations more agency in utilising resources and relaxed oversight measures. The state’s reliance on the sector highlights the importance of state funding, ensuring the sector is equipped to respond nimbly to emergencies. A secure third sector ensures that citizens have a safety net.

Although our respondents provided positive feedback about aspects of relationships between nonprofit organizations and government in Scotland, England, and Northern Ireland during the pandemic, the 2022 Scottish Government review expressed concerns about the ability of retaining such positive changes across sectors going forward. Furthermore, the disconnect or mismatch between policy expectations and outcomes, or policy rhetoric and practice reality – the so-called ‘implementation gap’ (Gunn 1978) – was highlighted by most respondents. This phenomenon in policy studies tries to explain failure in policy implementation, generally positioned as a fault of policy *implementors* rather than policymakers, and increasingly used as a ‘catch all’ concept for ineffective policy success and implementation at local or regional levels (Hudson, Hunter, and Peckham 2019). While disjuncture between policy rhetoric and reality where nonprofits are concerned is often pronounced (Mazzei and Roy 2017), the persistence of this ‘implementation gap’ idea perhaps indicates that governments across the UK do not always develop policies that are *implementable* in areas germane to nonprofits (such as those related to poverty, addressing social inclusion, and so on). It is clear from our study that a new way of doing politics and policymaking, which takes account of the highly complex, often hyper-localized, contexts in which many, if not most, nonprofits are embedded, is required.

Turning to our second question: In what ways do different policy contexts influence the work of nonprofit organizations, if at all? Our findings suggest that the devolved policy contexts did not always influence the day-to-day work of the non-profits across jurisdictions in clearly recognizable ways. An unexpected finding was that the overarching picture is exemplified by similarity across country level data rather than by difference. At first sight this is contrary to emerging evidence predicting that the policy divergence seen under devolution would have significant impacts on the day-to-day work of nonprofits (for example, as seen in the COVID response – see Acheson et al. 2022). But perhaps we overestimate the power of policy to shape the work of those nonprofits most rooted within their communities on a day-to-day level. And perhaps there is not *enough* policy divergence in the UK to see radically different practices at ‘street level’ between jurisdictions. Community-led nonprofits therefore seem to share consistent barriers and facilitators, regardless of policy divergence and jurisdiction, albeit we regularly see how successful partnerships between nonprofits and policymakers tend to be those that privilege the relational over the transactional, regardless of context (Lu 2015; but see also Brinkerhoff 2002).

Further evidence that some barriers and facilitators were independent of policy context was the similarity of issues in Northern Ireland with Scotland and England despite the lack of a stable working government there during the duration of this study. We emphasized from the outset of this paper the symbiotic nature of the relationship between government and the nonprofit sector, but nonprofits pragmatically get on with their ‘day job’ of supporting the communities they serve, in the absence of a sitting government (or at least politicians). Perhaps this is unsurprising given the roots of the nonprofit sector.

## 6 Conclusions

We set out to establish how policy acts as a barrier or facilitator to the work of community-led nonprofits concerned with place-based activities focused on improving the health and wellbeing of their immediate communities and the ways in which different policy contexts influence the work of nonprofit organizations, if at all. We have seen that policy can act both as barrier and facilitator in relation to a feeling of disconnection between policy and the realities of context; funding arrangements for nonprofits; tensions between the pursuit of mission and alignment to government priorities; in the implications for policy innovation; and in the relationships between nonprofits and policymakers and commissioners. However, our findings in relation to how differences in policy context influence the work of nonprofits in different ways are far less conspicuous.

There are probably at least two reasons for this lack of clear and unambiguous findings. The first explanation is that despite the presence of divergence in several areas of policy, the devolution settlement in the UK does not allow scope for *enough* policy divergence to provide for sufficiently different outcomes designed to cope with the entrenched spatial distribution of inequality and vulnerability that we see in the UK (Fransham et al. 2023). The design and implementation of highly localized responses that address the acute needs of communities, which support local nonprofits in such areas in unique ways, and which have clearly identifiable positive outcomes for communities, does not seem to happen to any great extent. The second explanation is that emergent ‘street level’ issues are, in fact, relatively similar across different jurisdictions and policy landscapes. Notwithstanding what was said earlier about how differences are likely to be highly constrained due to the nature of the devolution settlement in the UK, we did not find major differences in the day to day lives of non-profits between UK jurisdictions as seen from a ‘street level’ lens. Rather, where there is a relational disconnect between policymakers and nonprofits (e.g. poor communication/decision making; lack of co-production; poor personal contacts, and so on) these factors seem to cause the biggest ‘street level’ practice barriers. These do not seem to be unique to each jurisdiction but common across the different jurisdictions.

One of the main aspects we saw that influenced practice (positively) was the contextual factor of COVID-19, which saw a greater recognition for the interdependence between nonprofits and policymakers emerge in recognition of the shared goal of tackling the pandemic, often at a ‘hyperlocal’ level (Rendall et al. 2022). However, in the race to return to normal, clear and significant recognition of the role of nonprofits seems to have been passed over somewhat, at least in the UK. Relationships between nonprofits and government have returned to their pre-pandemic position where the power imbalance is clear and unambiguous, and with bureaucratic funding processes quickly being re-established. A ‘reset’ in the relationship between the nonprofit sector and government going forwards, which fully acknowledges and respects the role the sector played during the pandemic, has recently been called for (Roy, Bynner, and Teasdale 2023).

There are, of course, limitations to our study. There are not enough data to generalize experiences across nonprofits operating across the whole of the UK, but our work does provide an in-depth, and plausible account of views and experiences. Future research could include interviews with policy makers, and commissioners to reflect the views and experiences of people designing and delivering policies at the local, regional, and national levels. There was no attempt to achieve representativeness of each country, nor to account for significant differences within countries, and so the experiences of policy impacts on service delivery may differ greatly depending on location.

We have identified several policy implications for our study. The in-depth knowledge that nonprofits have of their communities can be beneficial for effective policy development if their involvement is valued and correctly resourced. Participants stated that barriers emerge when these are designed without consultation from those working directly with individuals experiencing the effects of these policies. Co-production should not feel like a ‘tick-box’ exercise, but an essential component of policy development, from the beginning of the design to the analysis of outcomes relating to funding, nonprofit service delivery, and the everyday realities of community members. Nonprofits should be provided with the necessary support to be fully engaged in the process of coproduction, including resources. And personal relationships matter a great deal to success.

Finally, the COVID-19 pandemic highlighted the importance of meaningful relationships between government and the nonprofit sector, and the positive impact of transferring power to community organizations to operate with much reduced bureaucratic oversight. This experience demonstrates that nonprofits can deliver quickly and effectively in times of crisis, but, with some imagination and a willingness by government to cede at least *some* power to nonprofit partners, such an approach could easily become the ‘new normal.’
